# Mechanical Dentistry

**Published:** 1873-01

**Authors:** E. S. Chisholm

**Affiliations:** Tuscumbia, Alabama


### Mechanical Dentistry.

BY E. S. CHISHOLM, TUSCUMBIA, ALABAMA.

*Read Before the Tennessee State Dental Society.*

Mechanical Dentistry, in its most general acceptation, comprises the construction of mechanical appliances as substitutes for the natural teeth. This alone does not constitute the limits of its meaning. It applies equally to the constructing of agents for the regulation of the natural organs, artificial palates, etc.

The substituting of artificial teeth for the natural ones was probably known long before the Christian era, though the exact time of its origin will probably ever remain in darkness. Hipprocrates, born four hundred years before Christ, alludes to the insertion of artificial teeth, fastened by gold wire. This art, however, remained in comparative obscurity up to the sixteenth century, when Eustachius flourished as an anatomist, and published perhaps the first work in dentistry. The art of printing having been introduced from this on, it received increasing attention. In the year 1774 the first porcelain teeth were introduced. The advantages of these over those carved from the bone and ivory, and the teeth of neat cattle, was discovered by a French apothecary; soon after which Chemant, a distinguished dentist of Paris, obtained a patent through Louis XVI. It was long after this before they came into general use. From this time forward knowledge in this specialty has been increasing in the mechanical as well as the operative department up to the introduction of cheap patent materials as substitutes for the finer metals in the former department. Since then my observation warrants the conclusion that it has been, and is gradually on the wane by the better members in our cause.

It was formerly passingly respectable for one to be a good mechanical operator, since this department required of him to be in every way familiar with the science of chemistry, metallurgy, and the collaterals, to this department, etc., but oh, how changed! Now mechanical dentistry gives the learned no advantage over the unlearned. These itinerating charlatans, who never spent one dollar or sacrificed a day's time to fit themselves for the profession, and who are able to pay for a right to use rubber, are estimated on a par by the populace with the student and practitioner who has devoted the youth of his life, and expended much of his hard earned means to prepare him for his calling. Like an incubus, the former visits his destructive influence by feeding from a profession which the latter may have expended years in building up.

As one has said, invention has given to dentistry no material more useful than vulcanite; none the use of which has proved more injurious. So dentistry has suffered its severest loss through its greatest gain.

Cheap materials bring common operations, since they are not sufficiently remunerative to the skillful. Their ease of manipulation places them within the reach of the uneducated pretender. Work characterized by comfort, durability, and artistic appearance, cannot so favorably impress the public as the show window exhibitions of high polish and regularity of arrangement, which probably possess not one element of these important essentials. This condition of things will not be improved until the people are educated by some means to discriminate the difference between good and bad operations. Hence the importance of the genuine operator marking his work with these distinguishing characteristics, and by no means allowing himself to be discouraged, that any branch of the cause may deteriorate.

But to draw more closely to the subject before us. The field of mechanical dentistry is so extensive in its limits, and of so much diversity, that I will ask to be pardoned for digressions, and failing to notice in regular order the various points in the construction of artificial dentures, since too much of this is common to us all. I will therefore mention a few practical points, which I have deemed worthy of notice.

The preparation of the mouth for artificial teeth is of no minor consideration. By no means should there be a sacrifice of any natural organs, unless they be few in number, and their remaining should materially effect the ultimate wearing of the artificial set. I will mention three or four such conditions.

1st. Should we find a few front teeth only remaining, and especially in the upper jaw, which are much abraded, antagonizing good teeth, their extraction would be indicated. The teeth to be substituted required to be of some length, and would be so short as to render them weak and unserviceable. Were you to make them longer they would not appear well, would discomfort the wearer in prehension and mastication since his custom was firmly in the use of the natural teeth.

2d. Where the entire lower set and upper molars are good, requiring the insertion of the front teeth. In such cases the molars are generally much worn, and when touching the lower, may bring the upper alveolus nearly, if not quite, in contact with the lower front teeth. It would here be better to extract the molars, and supply the whole upper jaw with teeth. In similar cases where the prominence of the superior alveolus is strictly parallel with the lower teeth, but the right or left side is higher than the other, is a great source of inconvenience to prevent the teeth appearing longer on one side.

3d. When the upper molars and bicuspids are to be supplied only, it is sometimes a matter of much difficulty, since the masticating on either side may break up the suction—the only means of support. The presence, too, of the front teeth, will greatly impair its sticking quality. If however, the gums are favorably shaped, and our patients persistent in wearing, success may be attained.

4th. If scattering teeth only in the front of the upper or lower jaw remain, it is better to remove, for these reasons:

1st. It is difficult to adjust the artificial gum accurately, so as to prevent detection.

2d. There is always some irregularity of position and direction of teeth, rendering the taking of a good impression difficult.

3d. Difference of color.

4th. The probability of their early loss in contact with the artificial.

Apart from these exceptions, I am in favor of restoring and preserving every natural tooth that our skill may possibly direct.

Since my experience is somewhat limited as to the new modes adopted as to time of insertion, I can say but little on this subject. My experience favors the awaiting the gums to heal, and a sufficiency of absorption as not to show any grove in the alveolar ridge, and until sharp processes disappear. I say this, duly recognizing the fact that suction arrests absorption, and pressure of friction increases it. Yet as most other rules it has its deviations. I have inserted plates upon newly healed gums, with good suction as permanent sets, that very soon, though patients wore night and day, required replacement. On the other hand, I have lost a number of permanent sets by the temporary remaining comfortable. I have also inserted teeth as soon after the removal of the natural as an impression could be taken, and teeth inserted, which are to-day comfortable. In one case the teeth were inserted as soon after the extracting as they could be made, but the mouth continued to swell for five days, when I inserted anew, with but little better result.

With these apparent contradictory indications the only way to reconcile them is to suppose that the same causes differently affect individuals influenced by age, temperament, and condition of health at the time. My experience admits the conclusion that in youth or health absorption is much more rapid.

Next in order we will consider briefly the taking of impressions. Formerly many materials were used for this purpose, and none were entirely subservient to the requirements. Yet plaster of Paris approximates more closely every exigency than any other known substance, and for this reason is in almost universal use. It is not, however, entirely free from objections; the most noticeable is its shrinking in process of crystalization. In using it for impressions I use salt water, which causes it to solidify much quicker. I use as little plaster as I can, having previously prepared a thin film of wax, attached to the posterior palatine border of the cup, to prevent plaster escaping posteriorly. In introducing the cup, previously directing patient to *hold the head erect, to be ready,* and when introduced *to breathe through the nose,* I would prefer my patient to be seated in a low chair. The cup when introduced should be pressed upon the posterior part and the anterior gently brought in place, which will press all surplus to the front, when sufficiently hard to remove.

When partial sets are to be substituted, it often becomes necessary to oil the impression cup first, and when sufficiently hard to break with a smart fracture, pull loose the cup, and with a suitable instrument cut the plaster so as to have it fracture, causing the least amount of breaking in its removal. These pieces may then be replaced in the cup with nearly a perfect adjustment.

Some little trouble having been experienced by many dental practitioners to prevent the rubber, when manipulating with this material, from pressing its way between the teeth or gums; we will mention *our* mode of *prevention*. The teeth should be ground proximally with their surfaces in contact. If any deviation from this, it should be in favor of their touching on their front or outer surfaces, giving the teeth the necessary proximal distance and inclination. Then with a round edged stone grind from the posterior of the gum a bevel of an angle of thirty degrees, so that the space which will be in contact with the rubber will form a V shape. Of course the grinding should not be sufficient to weaken the attachment of the pins, or to very nearly approximate the showing surface of the gum. Being then sunk in flask, and base plate removed, filled the spaces with oxychloride of zinc or plaster.

The artistic appearance of the teeth, when inserted, chiefly depends on what we term the "bite." It is here the length, size, and inclination are indicated. In this the operator's skill is taxed to the highest to rightly diagnose the deficiencies, and with an artistic eye supply the needed portion, arranging the teeth with studied negligence, and thus restoring the "*human face divine*." This is done, too, not disregarding the attributes of utility and comfort.

One of the greatest faults, with many young practitioners, is the arranging the teeth to describe a part of a circle which is very far from nature's order.

The cuspidate should be a little prominent, the bicuspids immediately behind them, with the outer cusps nearly in a direct line with the cusps of the canine and first molars.

This rule is not invariable, however. With some patients not near so much is required as with persons of round features, and broad face jaws.

The incisors of persons in strongly developed features may be a little longer and more prominent than the laterals.

As a general rule we too greatly fear the rendering conspicuous of our work. Some persons in earlier life unfortuately lose some of the natural teeth, which causes a dimunition of the jaw, when much prominence is necessary to restore the normal appearance.

Now, in conclusion, allow me to suggest to you, what will prove consoling to patients, and give them much satisfaction —be sure to inform them that work warranted always breaks —that work paid for always sticks.

